# Quantitative Imaging of Cholinergic Interneurons Reveals a Distinctive Spatial Organization and a Functional Gradient across the Mouse Striatum

**DOI:** 10.1371/journal.pone.0157682

**Published:** 2016-06-17

**Authors:** Miriam Matamales, Jürgen Götz, Jesus Bertran-Gonzalez

**Affiliations:** Clem Jones Centre for Ageing Dementia Research, Queensland Brain Institute, The University of Queensland, Brisbane, Queensland, Australia; Karolinska Inst, SWEDEN

## Abstract

Information processing in the striatum requires the postsynaptic integration of glutamatergic and dopaminergic signals, which are then relayed to the output nuclei of the basal ganglia to influence behavior. Although cellularly homogeneous in appearance, the striatum contains several rare interneuron populations which tightly modulate striatal function. Of these, cholinergic interneurons (CINs) have been recently shown to play a critical role in the control of reward-related learning; however how the striatal cholinergic network is functionally organized at the mesoscopic level and the way this organization influences striatal function remains poorly understood. Here, we systematically mapped and digitally reconstructed the entire ensemble of CINs in the mouse striatum and quantitatively assessed differences in densities, spatial arrangement and neuropil content across striatal functional territories. This approach demonstrated that the rostral portion of the striatum contained a higher concentration of CINs than the caudal striatum and that the cholinergic content in the core of the ventral striatum was significantly lower than in the rest of the regions. Additionally, statistical comparison of spatial point patterns in the striatal cholinergic ensemble revealed that only a minor portion of CINs (17%) aggregated into cluster and that they were predominantly organized in a random fashion. Furthermore, we used a fluorescence reporter to estimate the activity of over two thousand CINs in naïve mice and found that there was a decreasing gradient of CIN overall function along the dorsomedial-to-ventrolateral axis, which appeared to be independent of their propensity to aggregate within the striatum. Altogether this work suggests that the regulation of striatal function by acetylcholine across the striatum is highly heterogeneous, and that signals originating in external afferent systems may be principally determining the function of CINs in the striatum.

## Introduction

The striatum is a large subcortical structure involved in major cognitive and sensorimotor functions, which is regarded as the primary input to the basal ganglia network [1]. Lacking identifiable architectural feature or boundaries, the striatum is generally subdivided into functional subterritories on the basis of glutamatergic (mostly cortical and thalamic) and dopaminergic (from the midbrain) inputs [2]. In recent years, acetylcholine has been recognized as a major neuromodulatory signal in the striatum, which interacts closely with dopamine to integrate cortico- and thalamostriatal transmission [3]. The striatum hosts one of the most abundant sources of acetylcholine in the brain, largely supplied by local cholinergic interneurons (CINs) [4,5]. These neurons display a distinctive morphology (very large somata, aspiny dendrites and extensive axonal fields) and firing properties (tonic regular firing, long action potentials and large afterhyperpolarizations) [6–8], and their dysfunction has been implicated in Parkinson’s disease [9,10]. More recently, several studies have shown the emerging role of striatal cholinergic transmission in the processing of predictive information and the critical control of goal-directed action [11–14]. Therefore, establishing how the cholinergic interneuronal system contributes to striatal function depends on our ability to produce anatomically and physiologically realistic models of this local network. However, CINs make up a minor and spatially scattered fraction of the total neuronal population in the striatum (<3% in rodents), rendering the comprehension of the system’s connectivity a challenging task. The advent of novel tools that allow the identification of cell types through transgenic expression of fluorescent markers coupled with the imaging of large areas of tissue at high speed creates new opportunities for re-examining the architecture of this and other interneuronal systems. The present study aims at characterizing with unprecedented detail the anatomical and functional organization of the striatal cholinergic network by combining high-throughput fluorescence imaging, automated detection of neuron somata and quantitative assessment of CIN distribution across the different striatal subregions.

## Materials and Methods

### Data Availability Statement

The authors confirm that all data underlying the findings are fully available without restriction. All data files are available from the data repository figshare (https://dx.doi.org/10.6084/m9.figshare.3420961.v1).

### Ethics Statement

This research has been performed in agreement with the *Australian Code for the Responsible Conduct of Research* (National Health and Medical Research Council, Australian Research Council and Universities Australia, 2007). All experimental procedures were approved by the University of Queensland Animal Ethics Committee (approval numbers: 027–12 and 327–11), and were conducted in accordance with the *Animal Care and Protection Regulation* (Queensland Government, 2012) and the *Australian Code of Practice for the Care and Use of Animals for Scientific Purposes* (National Health and Medical Research Council, 2014).

### Animals

Four two-month-old male wild-type C57Bl/6 mice and four two-month-old male homozygous *ChAT*^BAC^-EGFP transgenic mice (http://jaxmice.jax.org/strain/007902.html) were used in this study [15,16]. Animals were maintained on a 12 h light/dark cycle with food and water provided *ad libitum* and were kept with their littermates until used for the experimental procedures described below. Experiments were carried out during day time.

### Immunofluorescence

Naïve mice were taken from their home cage and rapidly anaesthetized through exposure to isoflurane (4% in air) in a sealed chamber followed by an injection of sodium pentobarbital (350 mg/kg i.p.), and transcardial perfusion with 4% paraformaldehyde in 0.1 M sodium phosphate buffer (pH 7.5) using an air pressure system (constant flow of 15 ml/min). Vibratome-cut free-floating coronal sections were processed for immunofluorescence staining as described previously [17]. Briefly, sections were rinsed in washing buffer (0.25 M Tris, 0.5 M NaCl, 0.1 mM NaF, pH 7.5). Following tissue permeabilization in 0.2% Triton X-100, sections were incubated overnight at 4°C with primary antibodies: anti-choline acetyltransferase (ChAT; 1:500; Millipore; #AB144P); anti-EGFP (1:500; Life Technologies, #A11122); anti-phosphorylated ribosomal protein S6 (p-rpS6; 1:300; Cell Signaling Technology, #2215). Unbound primary antibodies were washed off and sections were incubated for 60 min at room temperature with the following fluorescent dye-conjugated secondary antibodies (Jackson ImmunoResearch Laboratories): Cy3 donkey anti-goat (1:800; #705-165-003); Cy3 donkey anti-rabbit (1:800; #711-165-152); Alexa Fluor 488 donkey anti-rabbit (1:800; #711-545-152). Sections were then rinsed three times and nuclei were counterstained with 1 μg/mL DAPI (Life Technologies) for 5 min at room temperature. After a final wash, sections were mounted on Superfrost Plus coated slides (Thermo Fisher Scientific) with Vectashield fluorescence medium (Vector laboratories) and a coverslip was mounted and sealed.

### Image Processing and Analysis

#### Analysis of CIN population

We obtained 102 serial sections (bregma: 1.98 to -1.58 mm, 30 μm-thick) from a *ChAT*^BAC^-EGFP mouse and imaged them using a Metafer slide scanning system (MetaSystems). Images were processed using ImageJ2/Fiji (v.1.49k, Wayne Rasband, National Institutes of Health). The outer boundary of the right striatum and its anatomical subdivisions (dorsal portion of the anterior striatum (aDS), shell (ShVS) and core (CoVS) of the ventral striatum, dorsomedial (pDMS) and dorsolateral (pDLS) areas of the posterior striatum) were manually drawn in accordance with a mouse brain atlas [18], and the area (mm^2^) and Cartesian coordinates of the points defining each region’s edge were obtained. Images were then thresholded based on EGFP intensity levels and individual CIN somata (EGFP positive) were automatically detected using the “Analyze Particle” command. A table containing the Cartesian coordinates (*x*, y) of the centroid was obtained.

The spatial distribution of all detected CINs was digitally reconstructed using a customized program in MATLAB (The MathWorks). The total count and density (CINs/mm^2^) of CINs was calculated for each striatal territory. The same data files were used to assess CIN spatial organization using the DBSCAN (density-based spatial clustering of applications with noise) clustering algorithm in MATLAB. For all analyzed sections, we set the parameters for DBSCAN algorithm considering a point to be included within a cluster when it had at least six neighboring points located within a circle of 150 μm radius. Finally, we calculated the nearest neighbor distance of CINs using a customized triangulation-based algorithm in MATLAB. Finally, a custom written program in MATLAB [19] was used to quantify and visualize the clustering of CINs using Ripley’s *K*-function [20,21] and to calculate 95% Monte Carlo confidence intervals by calculating Ripley’s *K*-function from simulated point maps (500 simulations/section) containing the same number of CINs at a given area for each striatal section analyzed [22]. The *L*-function was used to test whether the distribution of points within a concentric circle of radius *r* obeys complete spatial randomness, in which case *L*(*r*)–*r* will be zero. If at some radius the value of *L*(r)–r is positive, it means that more points have been encircled at that radius than would have been expected for a random distribution, i.e. the points are clustered. Cluster maps were generated from Ripley’s *K*-function in which each dot corresponds to one CIN, which was pseudo-colored according to its individual *L(r)* value calculated for a 200-μm radius. We chose *r* = 200 μm to represent clustering in each section because at that radius, the *L(r)* value calculated from the Ripley’s *K*-function peaked in most of our samples.

#### Network reconstruction

We used 20 μm-thick sections from three *ChAT*^BAC^-EGFP transgenic mice (bregma: 1.10 and 0.26 mm) for the digitization and analysis of striatal cholinergic global neuronal branching. High magnification images were acquired with a LSM 510 META confocal laser-scanning microscope (Carl Zeiss). Serial optical sections along the *z* axis were obtained from each striatal subregion (3 *z*-stacks per subregion composed of 70–100 images). Analysis of global spatial complexity of the striatal cholinergic network was performed using NeuronStudio [23]. Data regarding the total branching (μm) and the number of branch points (junctions) were retrieved.

#### Fluorescence analysis of cholinergic and activity markers

Five sections (bregma: 1.42, 1.1, 0.62, 0.14 and -0.22 mm) from four C57Bl/6 mice were co-stained with antibodies against ChAT and p-rpS6 and imaged using the Metafer slide scanning system. The location of 2,107 CINs was recorded as described above and a region of interest (ROI) of the contour of individual somata was obtained and overlaid onto the p-rpS6 image to measure the fluorescence intensity (mean gray value, m.g.v) for individual neurons.

### Statistical Analysis

Data were analyzed using SPSS Statistics software (version 20, IBM Corporation). The *a priori* alpha level was set at *p* < .05. One-way analysis of variance (ANOVA) was conducted followed by Tukey’s (HSD) multiple comparisons test when the variances were equal across groups (Levene’s test, *p* > .05). However, when the assumption of homogeneity of variances was violated (Levene’s test, *p* < .05), a Welch’s ANOVA correction was applied together with the Games-Howell *post hoc* test. To measure the relationship between p-rpS6 intensity and distance to the most dorsomedial point, Pearson’s correlation coefficient (*r*) and its significant level were calculated.

## Results

### Cholinergic Interneurons Are Differentially Distributed across the Mouse Striatum

**Fig 1:**
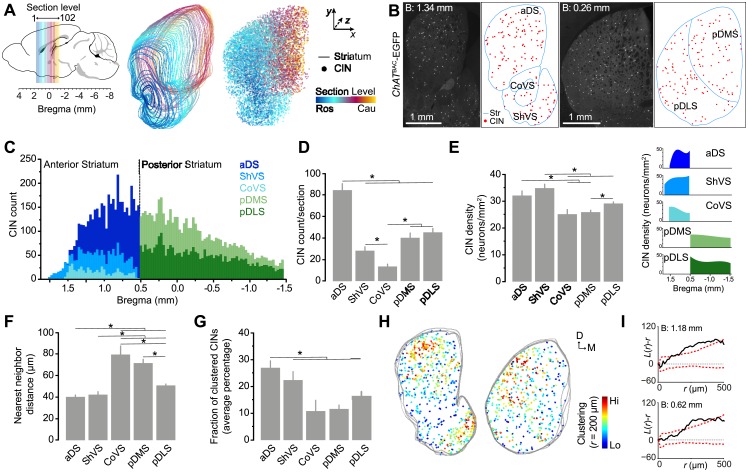
Spatial distribution of cholinergic interneurons in the mouse striatum. (A) Stereological reconstruction of CINs in the right striatum of a *ChAT*^BAC^-EGFP mouse brain. Colored lines demarcate the outer boundary of the striatum at overlapping sections; individual dots correspond to the cell bodies of mapped CINs within each section. Color code indicates their location along the rostrocaudal (z) axis. (B) Images of single coronal sections of the anterior (left) and posterior (right) striatum (bregma 1.34 and 0.26 mm, respectively) of a *ChAT*^BAC^-EGFP mouse and corresponding digital reconstructions. The outer boundaries of striatal subdivisions are outlined in blue; red dots are detected CINs. (C) Stacked bar graph showing the distribution of CINs in the different striatal subregions for individual serial sections along the rostrocaudal axis (N = 102 sections). (D) Mean of total identified CINs per hemisection (+1 S.E.M, N = 40 anterior and 63 posterior sections). (E) Mean of region-specific densities (+1 S.E.M, N = 40 anterior and 63 posterior sections). Right insets correspond to CIN density for individual sections along the rostrocaudal extent. (F) Mean of nearest neighbor distance of detected CINs in the different striatal territories (+1 S.E.M, N = 40 anterior sections and 63 posterior sections). (G) Average percentage (+1 S.E.M, N = 29 anterior and 39 posterior sections) of CINs located within clusters relative to total CINs per section. (H) Cluster maps generated from Ripley’s *K*-function from three anterior (left) and three posterior (right) consecutive coronal sections. Each dot represents one CIN pseudo-colored with its value of *L(r)* at a 200-μm radius. (I) Representative Ripley’s *K-*function analysis of the CIN coordinates obtained from an anterior (1.18 mm bregma) and a posterior (0.62 mm bregma) striatal section. Black solid line plots *L(r)*–*r* function for observed CIN distributions in an individual section (equals 0 when complete randomness) and dashed red lines plots 95% confidence intervals that were calculated from 500 spatially random simulated distributions. Asterisks denote significant effect (see *p* values in text).

To analyze the spatial organization of the striatal cholinergic system, we digitally reconstructed 102 serial coronal sections spanning the rostrocaudal extent of the right striatum from a *ChAT*^BAC^-EGFP mouse, and mapped the position of 9,732 fluorescently labeled CINs (Fig 1A). We next reconstructed their regional distribution in the following anatomical subdivisions: dorsal portion of the anterior striatum (aDS), the shell (ShVS) and core (CoVS) of the ventral striatum, and the dorsomedial (pDMS) and dorsolateral (pDLS) areas of the posterior striatum (Fig 1B). The rostrocaudal profile of total cell counts showed that CIN numbers were highest in the middle striatum (approximately at 0.8 mm from bregma) and progressively declined caudally (Fig 1C). When classifying CIN location based on striatal subterritories, we found that in dorsal regions the average CIN counts per hemisection was twofold higher in the aDS than in the pDMS and pDLS (*p* < .001), and in ventral regions, the average in the ShVS was also twofold higher than in the CoVS (*p* = .039) (Fig 1D). Overall, the CoVS contained significantly less CINs than any other striatal region (*p* < .05).

We next examined the average density of CINs and found that it significantly varied across striatal subdivisions (*F*_(4, 88.34)_ = 10.475, *p* < .001) (Fig 1E). We observed that CINs were most densely packed in the ShVS, which displayed 1.5-fold higher average density than the CoVS (*p* < .001), pDMS (*p* < .001) and pDLS (*p* = .004). The aDS also contained significantly greater concentration of CIN when compared to the pDMS (*p* = .012), which suggests a rostrocaudal gradient of CIN density. Furthermore, within the ShVS, there was a gradual two-fold increase of density along the rostrocaudal axis (from 20 to 47 CINs/mm^2^), whereas in the CoVS we observed a two-fold decrease density from anterior to most posterior levels (from 29 to 12 CINs/mm^2^) (Fig 1E, inset). The overall CIN density did not vary considerably along the rostrocaudal axis within the pDMS and pDLS.

To further investigate the spatial pattern of CINs in the striatum, we conducted a nearest neighbor distance analysis which revealed strong differences in CIN distributions between striatal subregions (*F*_(4, 90.468)_ = 24.957, *p* < .001) (Fig 1F). In line with our density analysis, distances between CINs were significantly shorter in high-density sectors (i.e., aDS and ShVS) compared to low-density sectors (i.e, CoVS and pDMS; *p* < .001). Next, we considered whether CINs form spatially organized clusters, as other interneuronal populations do in the mouse neocortex and the spinal cord [24,25]. We first performed a density-based cluster analysis of neuronal distribution, in which we defined a group of neurons as a cluster when at least 6 CINs were found within a 150 μm radius. This analysis revealed that the vast majority of CINs do not cluster together, with only 17% of the detected CINs being located into clusters. We then calculated the proportion of CINs forming cluster in each striatal subregion and found that the aDS is the region in which CINs tend to aggregate in a higher degree, with an average of 26% of total neurons being located within identified clusters (*p* < .05 vs CoVS, pDMS and pDLS). There was not significant difference in the propensity of clustering between the other striatal sectors (Fig 1G).

In order to determine if these spatial arrangements were observed more often than expected from complete randomness, we used a cluster-analysis method based on Ripley’s *K*-function [20,21] and calculated 95% Monte Carlo confidence intervals from randomly simulated data points for each section (500 simulations/section). This analysis showed that over 40% of the coronal sections analyzed along the rostrocaudal axis (31 out of 76 sections in total) exhibited a discrete though significant clustering of CINs across the striatal tissue (Fig 1H and 1I). Taken together, these findings show that, even though CINs appear to distribute sparsely throughout the striatum, they have region-specific tendency to establish neuronal aggregates, which may influence their functional connectivity.

### Digital Reconstruction of Striatal Cholinergic Networks

**Fig 2:**
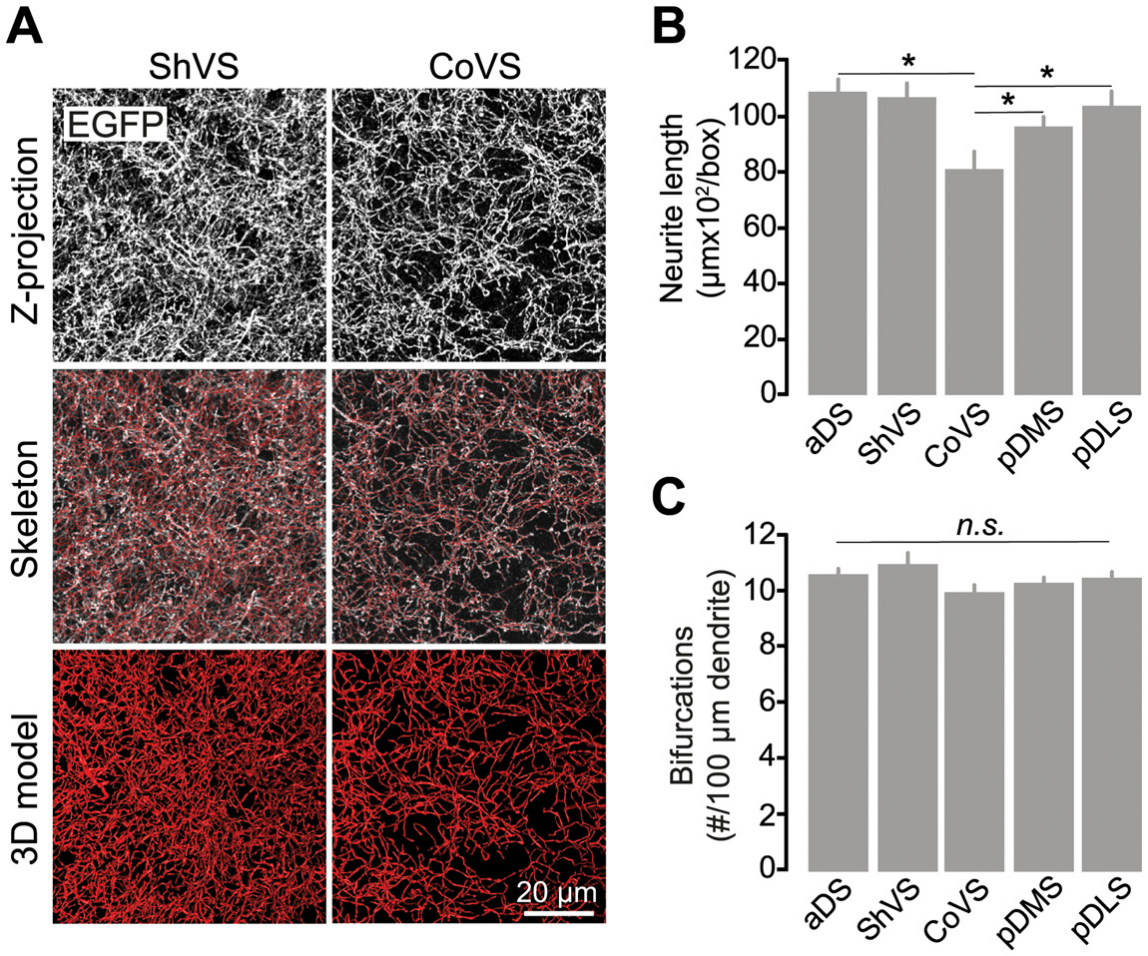
Quantitative characterization of cholinergic neuropil in striatal regions. (A) Representative three-dimensional reconstruction of cholinergic dendritic arbors in the ShVS and CoVS of a *ChAT*^BAC^-EGFP mouse. (B) Mean (+1 S.E.M) of the overall cholinergic neurite length in a 100-μm^3^ volume of striatal tissue. (C) Mean (+1 S.E.M) of the number of bifurcations per 100-μm length of cholinergic dendrite. N = 3 mice; 3 volumes/sector. Asterisks denote significant effect (see *p* values in text). *n*.*s*., nonsignificant.

We next sought to assess differences in the global structure of the CIN network across distinct striatal subterritories in three *ChAT*^BAC^-EGFP mice. We reconstructed the cholinergic multineuron network contained in a ~100 μm^3^ box of the different striatal sectors (3 volumes/region) with similar neuronal density and assessed their spatial complexity through automated quantitative methods (Fig 2A). We first analyzed the extent of CIN arborization and found that the overall dendritic length was significantly different across striatal subdivisions (*F*_(4, 40)_ = 6.676, *p* < .001). The total content of cholinergic neuropil in the CoVS was significantly shorter compared to the aDS (*p* = .001), the ShVS (*p* = .002) and the pDLS (*p* = .007), but not the pDMS (*p* = .170) (Fig 2B). Furthermore, examination of CIN neuropil complexity, as measured by the number of arbor bifurcations per 100 μm of dendrite, did not reveal overall statistically significant differences between striatal regions (*F*_(4, 19.749)_ = 2.163, *p* = .111) (Fig 2C). These data therefore show that, while cholinergic neuropil is homogeneously compact throughout dorsal territories and the ShVS, its density is relatively scarce in the CoVS, although branching patterns in cholinergic ramifications are constant in all striatal subdomains.

### Cholinergic Interneurons Display Region-Specific Baseline Activity

**Fig 3:**
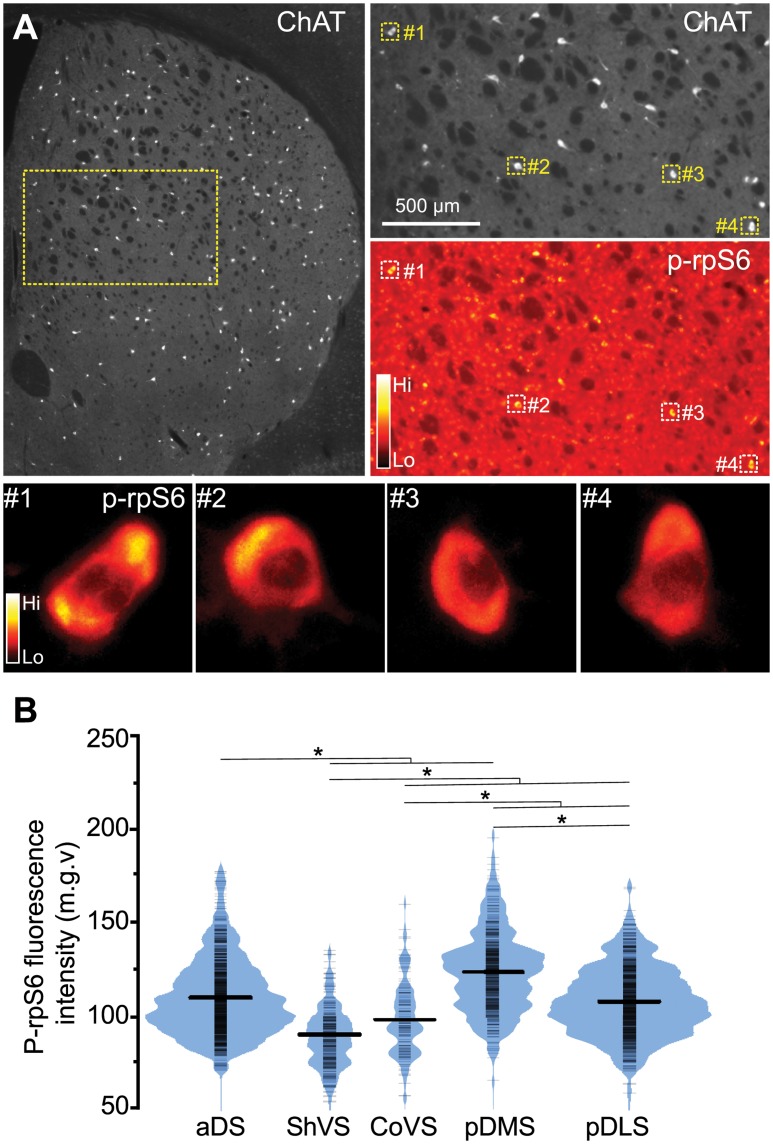
Cholinergic interneurons display region-specific gradients of activity. (A) Low magnification image of a mouse section double-stained with ChAT and p-rpS6 antibodies. Right panels show ChAT (top) and p-rpS6 (bottom) staining in CINs with four squares indicating CINs that are shown in a magnified view at the bottom. Bottom panels show p-rpS6 labeling visualized with the “Orange Hot” look-up table to highlight the fluorescence intensity. (B) Bean plot showing p-rpS6 signal (mean gray value) of CINs detected in the different striatal territories (N = 2,107 neurons, 4 mice). Short strokes (in black) indicate fluorescence values of individual neurons and long strokes the mean value. Colored curves represent the frequency distribution of neurons for each fluorescence intensity value. Asterisks denote significant effect (see *p* values in text).

Cholinergic interneurons (presumed tonically active neurons [TANs]) typically display a tonic pattern of firing that is maintained even if all their synaptic inputs are blocked [7,17]. Recently, we developed an immunofluorescence-based method that allows to visualize this baseline tonicity and to estimate broad changes in the activity of large numbers of CINs, which relies on detection of phosphorylated ribosomal protein S6 (p-rpS6) in individual neurons throughout the striatal tissue [11,14,17]. Here, in order to assess whether regional differences in CIN activity were observable across the different territories of the striatum, we quantified p-rpS6 immunofluorescence levels in over 2,000 ChAT-positive neurons from naïve animals (4 mice, 5 sections/mouse) (Fig 3A).

Interestingly, we found that CIN p-rpS6 fluorescence intensity varied significantly among striatal subdivisions (*F*_(4, 631.085)_ = 139.023, *p* < .001) (Fig 3B). In the anterior areas, aDS displayed the highest p-rpS6 intensity compared to ventral regions (*p* < .001). We also found that the degree of rpS6 phosphorylation in the ShVS was significantly lower than in the CoVS (*p* < .001). In regions of the posterior striatum, CINs in the pDMS displayed higher levels of p-rpS6 signal compared to those located in the pDLS (*p* < .001). These findings reveal that CINs residing in different striatal subdivisions display particular levels of intrinsic cellular activity in naïve animals, which raises the possibility that functional cholinergic gradients may exist throughout the striatal tissue in basal conditions.

### Cholinergic Interneurons Show a Dorsomedial-to-Ventrolateral Gradient of Activity in the Striatum

**Fig 4:**
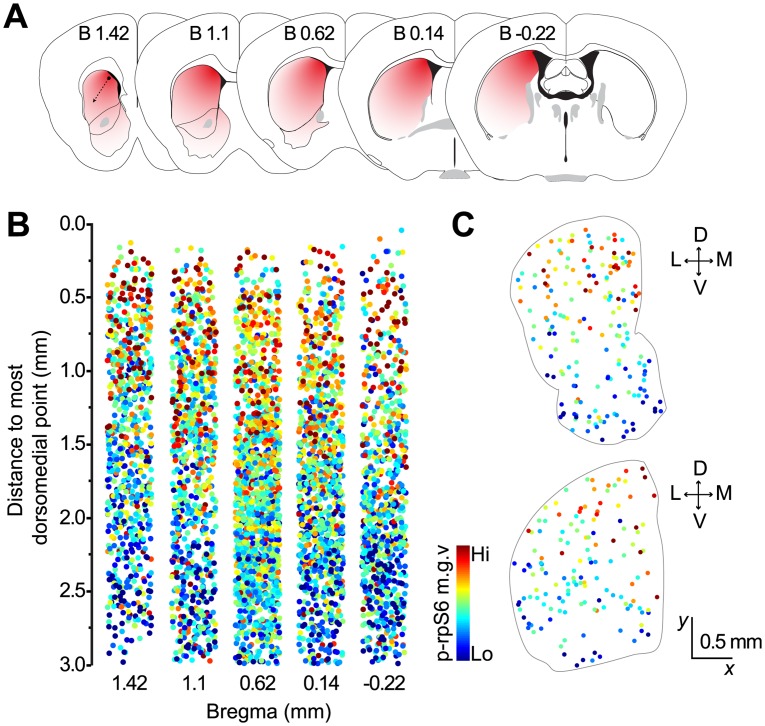
Downward gradient in S6rp phosphorylation levels in CINs along the dorsomedial-to-ventrolateral axis. (A) Coronal mouse brain diagrams at the levels analyzed illustrating a red-to-white color gradient from the most dorsomedial point of the striatum (dashed arrow). (B) Quantification of p-rpS6 in individual CINs (color-coded dots) and their distance to the most dorsomedial point. (N = 2,107 neurons, 4 mice). (C) Spatial mapping of CINs color-coded according to their relative p-rpS6 fluorescence levels in representative sections of the anterior (top) and posterior (bottom) striatum.

It is well known that cortical and thalamic projections to the striatum distribute along a dorsomedial-to-ventrolateral zone, forming a gradient of excitatory inputs [26,27]. Therefore, we next sought to assess whether the differences we observed in CIN baseline conditions across the striatum (as per p-rpS6 fluorescence) adhere to the functional patterns presumably established by striatal afferent connections, as opposed to a strict organization based on classic anatomical subdivisions. To test this, we co-labeled five mouse coronal sections at different rostrocaudal levels with p-rpS6 and ChAT, and measured the distance of each identified CIN to the most dorsomedial point of the striatum (Fig 4A). We found that, for all rostrocaudal levels analyzed, the cellular activity of CINs was highest in dorsomedial areas and gradually decreased toward ventrolateral regions, following a functional gradient similar to the one established by corticostriatal afferents [28] (Fig 4B and 4C). Statistical analysis supported these observations, revealing a strong correlation between p-rpS6 levels in individual CINs and their distance to the most dorsomedial point (B 1.42 mm: Pearson’s *r* = -.439; B 1.1 mm: *r* = -.371; B 0.62 mm: *r* = -.390; B 0.14 mm: *r* = -.386; B -0.22 mm: *r* = -.396; *p* < .0001 for all levels analyzed). Taken together, these results suggest that the topographical organization of excitatory cortical and thalamic synaptic inputs to the striatum, and probably others, might play a role in the region-specific differences observed in striatal CIN intrinsic activity in naïve animals.

## Discussion

In this study, we employed a quantitative imaging approach to analyze the intrinsic architecture of the entire CIN population in a fully reconstructed mouse striatum and to assess overall CIN activity levels throughout this brain region. Expanding previous observations in rodents and primates with greater detail and accuracy, we found that the rostral striatum contained a higher concentration of CINs than the caudal striatum, a gradient that has also been observed in the rat striatum [29–31]. The current results provide clear evidence that CINs distribute heterogeneously in the distinct anatomical subdivisions of the striatum, likely providing a differential, region-specific, cholinergic modulation to striatal projection neurons [32–34]. This uneven topographical organization was remarkable in the ventral striatum, with the shell containing greater cholinergic innervation than the core, which also contained a less dense cholinergic neuropil. These findings extend previous research in rats and primates [35,36], and suggest that information processing in ventral striatal subregions may involve different cholinergic environments.

Although it is generally assumed that CINs are dispersed in a random manner throughout the striatum, the novel visualization and analytical tools employed in the current study enabled us to determine this observation quantitatively. The different spatial point pattern statistics that we applied to our neuronal dataset revealed that the proportion of detected CINs arranged into cluster was low (17%), but there was however significant clustering of CINs along the rostrocaudal extent of the striatum, particulary in the anterior dorsal striatum and the shell of the ventral striatum. Interestingly, the observation that these clusters were confined to several consecutive sections in the two-dimensional space raises the possibility that CINs may establish local functional ensembles, or microcircuits, within the striatal network. Indeed, it is likely that future neuroanatomic studies addressing the three-dimensional organization of this interneuronal population using non-sectioned tissue of the whole striatum, will find a higher degree of clustering than what we could observe here [37,38]. The spatial patterning of CINs in the anterior striatum differs from that found for striatal projection neurons (the largest population of neurons in the striatum) in that these appear to follow complete spatial randomness in rostral areas [39]. The current findings provide support for a region-specific organization of CINs, but whether and how this arrangement influences the specific modulation of striatal function by acetylcholine in the distinct striatal subterritories needs to be further investigated [3].

Of particular interest are our findings in naïve animals that baseline intrinsic activity of CINs varied depending on their location within the striatal tissue. CINs have been extensively studied electrophysiologically and can be reliably identified based on their distinctive morphological features and membrane properties [7,8]. However, limitations inherent to electrophysiological recordings do not allow for a comprehensive stereological assessment necessary to address whether CIN cellular responses change throughout the entire striatal tissue. In the present study, we used p-rpS6 as a surrogate marker of CIN activity, which has been shown to follow the spiking pattern of CINs under basal and stimulated conditions [11,17]. We do not intend to link the firing of these neurons, occurring in milliseconds, to a fluorescence-based biochemical signal which likely reflects the sum of the activity of the neuron over many minutes. Nevertheless, our quantitative fluorescence analysis of over 2,000 CINs allowed us to estimate broad differences in baseline activity in this minor neuronal population of the striatum (<3%), which would be unachievable with classic electrophysiological profiling. Using this method, we found that CIN’s baseline activity levels greatly vary among striatal subdivisions: p-rpS6 signal was significantly lower in ventral and lateral regions, and gradually increased toward dorsomedial territories. Along this line, previous studies comparing the neuronal activity of tonically active neurons (presumed CINs) in monkeys during the performance of a behavioral task found that their response profiles were dependent on the location within a particular striatal territory [40]. Together, these findings suggest that the position of CIN distinguishes different physiological cholinergic states in the striatum and raises the question of how the differential activity of CINs is established throughout the striatal network.

It is well known that the function of CINs is largely influenced by excitatory thalamic and cortical inputs, whose glutamatergic terminals establish decreasing density gradients along the dorsomedial-to-ventrolateral and the medial oblique axes, respectively [26,28,41]. In the present study, we also found a decreasing gradient of CIN activity along the dorsomedial-to-ventrolateral axis. It is therefore reasonable to speculate that the differences observed in CIN activity levels could depend on the origin of their glutamatergic inputs. Additionally, the topographical arrangement of other afferents to the striatum, such as dopaminergic inputs, might also differently influence the excitability of CINs. In this context, recent work in our laboratory has shown that deterioration of a thalamic-to-cholinergic interneuron pathway due to normal aging involves altered firing in CINs with a reduced propensity to undergo burst-firing, together with a decrease in p-rpS6 signal in CINs of the posterior striatum of aged mice as compared to young ones [14]. Taken together, evidence suggests that the spatial location of a CIN within the striatal tissue will establish its connectivity, and thus determine the role it plays in the overall function of the striatal system.

An additional level of complexity regarding the functional organization of the striatum is its segregation into patch (striosome) and matrix compartments [42]. This classification of the striatal territory is made on the basis of specific neurochemical markers and the differential distribution of cortical afferents [43]. The distribution of CINs in relation to patch and matrix compartments of the striatum has been previously described in cat, monkey and rat [44,45]. These reports have shown that in all the species studied, cholinergic neuropil is concentrated in the matrix and is weak in the patch compartment whereas cell bodies are principally located within the matrix compartment. Whether this compartmental organization also influences the activity profile of CINs remains to be explored.

The functional investigation of neuronal circuits and systems is a fast-growing area in neuroscience, and it requires detailed information on the spatial organization and connectivity of full neuronal networks within the brain. In this study we have undertaken an imaging approach that provides an exhaustive representation of the distribution and baseline activity of a large number of cholinergic interneurons throughout the striatum of the mouse, thus expanding our understanding of the striatal cholinergic network, a critical system within the basal ganglia. The comprehensive data generated from this and other neuroanatomical studies, combined with genetic approaches for specific neuronal activity monitoring and manipulation, should contribute to generate a realistic functional connectome of the striatum, which will help deepen our knowledge of basal ganglia function.
